# NMR-based metabolomics in pediatric drug resistant epilepsy – preliminary results

**DOI:** 10.1038/s41598-019-51337-z

**Published:** 2019-10-21

**Authors:** Łukasz Boguszewicz, Ewa Jamroz, Mateusz Ciszek, Ewa Emich-Widera, Marek Kijonka, Tomasz Banasik, Agnieszka Skorupa, Maria Sokół

**Affiliations:** 1 Department of Medical Physics, Maria Sklodowska-Curie Institute - Oncology Center, Gliwice Branch, Wybrzeże Armii Krajowej 15, 44-101 Gliwice, Poland; 2 Department of Pediatric and Neurology of Developmental Age: The Independent Public Clinical Hospital No6 of Medical University of Silesia Katowice, Katowice, Poland; 3 Department of Pediatric Neurology School of Medicine in Katowice, Medical University of Silesia Katowice, Katowice, Poland

**Keywords:** Epilepsy, Biological physics

## Abstract

Epilepsy in children is the most frequent, heterogeneous and difficult to classify chronic neurologic condition with the etiology found in 35–40% of patients. Our aim is to detect the metabolic differences between the epileptic children and the children with no neurological abnormalities in order to define the metabolic background for therapy monitoring. The studied group included 28 epilepsy patients (median age 12 months) examined with a diagnostic protocol including EEG, videoEEG, 24-hour-EEG, tests for inborn errors of metabolism, chromosomal analysis and molecular study. The reference group consisted of 20 patients (median age 20 months) with no neurological symptoms, no development delay nor chronic diseases. ^1^H-NMR serum spectra were acquired on 400 MHz spectrometer and analyzed using multivariate and univariate approach with the application of correction for age variation. The epilepsy group was characterized by increased levels of serum N-acetyl-glycoproteins, lactate, creatine, glycine and lipids, whereas the levels of citrate were decreased as compared to the reference group. Choline, lactate, formate and dimethylsulfone were significantly correlated with age. NMR-based metabolomics could provide information on the dynamic metabolic processes in drug-resistant epilepsy yielding not only disease-specific biomarkers but also profound insights into the disease course, treatment effects or drug toxicity.

## Introduction

Based on the official International League against Epilepsy (ILAE) report, Fisher *et al*. proposed the following clinical definition of epilepsy embracing occurrence of any of the following conditions: - at least two unprovoked seizures which occurred >24 hours apart; - one unprovoked (or reflex) seizure together with a probability of further seizures similar to the general recurrence risk (at least 60%) after two unprovoked seizures, taking place over the next 10 years; - diagnosis of an epilepsy syndrome^1^

Being the most frequent chronic neurologic condition in childhood, epilepsy afflicts around 1% of children. During the first 10 years of life 1 out of 150 children is diagnosed with epilepsy, with the highest incidence rate observed during infancy^2^.

The etiology of epilepsy is found in 35–40% of patients. Large heterogeneity of epileptic syndromes and the pleiotropic effect of genes make classification of epilepsy difficult. Moreover, the research shows that between 6% and 41% patients, does not respond in an adequate manner to antiepileptic drugs (AEDs) and their disease evolve into refractory (intractable) epilepsy^3,4^.

In 2017, the ILAE released an update of classification of seizures and the epilepsies^5–8^. The diagnostic procedure involves the seizure-type diagnosis (focal, onset, generalized onset, unknown onset) the epilepsy-types diagnosis (focal epilepsy, generalized epilepsy, combined generalized and focal epilepsy, or unknown epilepsy types) and recognizing epilepsy syndromes^1,5–7^. Among the poor prognosis factors in epilepsy a younger onset and a longer epilepsy duration are being mentioned. As reveals from the Ramos-Lizana *et al*. study^3^ the worse prognosis is expected in children below 12 months old suffering from symptomatic epilepsy accompanied with pathological neuroimaging study, or with frequent seizures before diagnosis of drug-resistant epilepsy. Another strong factors indicating poor prognosis are reported by Sillanpaa & Schmidt^9^ and include seizures with weekly frequency during the first year of treatment or prior to treatment as well as diagnosis of remote symptomatic epilepsy.

Though magnetic resonance imaging (MRI) is an appropriate tool to be applied to identify an epilepsy cause (e.g., tumors, traumatic brain injury, vascular malformations, hippocampal sclerosis, cortical malformations), in nearly 30% of cases there are no clear epileptogenic lesions (non-lesional or MRI-negative)^10^. *In vivo* proton magnetic resonance spectroscopy (1H-MRS) is helpful in localization or lateralization of the epileptogenic foci and in the patient monitoring during the antiepileptic therapy and/or after resection of the epileptogenic tissue. However, in case of the patients with non-lesional insular epilepsy the spectroscopic method is at least inadequate. Because there are only several lines present in the *in vivo* MR spectra - it is impossible to identify the molecular paths and find the real biomarkers from such spectra. The brain metabolites being disturbed are primarily N-acetyl aspartate (NAA), creatine (Cr) and phosphocreatine (PCr), cholines (Cho), glutamine (Gln) and glutamate (Glu)^11–14^. An additional difficulty is the fact that most of the works concern the epilepsy in adults, whereas, there is little data on the use of magnetic resonance spectroscopy in the studies of drug-resistant epilepsy in children. The latter, though valuable, comprise rather small and heterogeneous populations^14,15^.

High resolution *in vitro* NMR (HR NMR) spectroscopy of body fluids is more feasible for pediatric patients and gives insight into a significantly larger (about ten times) group of detectable metabolites. Application of multivariate projection methods to analyze the spectroscopic data allows for examination of the metabolome (i.e. detection of the low molecular weight metabolites present in a particular biological system: fluid, cells or tissue), to determine its changes and mutual correlations of the individual metabolites. Such approach to the studies of metabolism – called metabolomics^16^ – allows for better insight into the biological basis of drug resistance in epilepsy. Because metabolism is, an important regulator of neuronal excitability, thus its dysregulation after a seizure, may lead to many damaging consequences. The influence of the “disturbed metabolism” (which is, however, under control) is seen in the ketone diet – such diet is being applied to reduce or prevent seizures in children with drug-resistant epilepsy^17^.

Thus, serum 1H NMR based metabolomics approach seems to be more suitable to detect the metabolic signatures in epilepsy, to predict disease state and response to treatment. Serum as a systemic pool of metabolites, reflects all processes and their systematic disturbances, even those in the brain due to the bi-directional flow of endogenous metabolites between blood and brain compartments.

The central hypothesis of this work is that epilepsy has a unique chemical signature influencing the naturally occurring chemicals and metabolic pathways in the brain. Thus, our aim is to detect the metabolic differences between the epileptic children and the children with no neurological abnormalities in order to define the metabolic background for therapy monitoring.

## Materials and Methods

### Subjects selection

The study was approved by the ethical committee of the Silesian Medical University, and written informed consent was obtained from each child’s parent(s) or guardian to the participation of their child in the study. All methods were performed in accordance with the relevant guidelines and regulations.

48 patients were enrolled in the study. The patients were divided into two groups: the Epilepsy Group (EG) and the Reference Group (RG). EG included 28 individuals suffering from epilepsy (median age was 12 months, female to male ratio was 13:15). The seizure types, epilepsy types and epilepsy syndromes were defined according to the International League Against Epilepsy Classification and Terminology (ILAE 2017). All children with epilepsy (EG) underwent diagnostic protocol including EEG, videoEEG, 24-hour EEG (in selected sapienti), laboratory tests for inborn errors of metabolism, chromosomal analysis and molecular study. All patients with epilepsy were treated with 1 to 5 antiepileptic drugs (AEDs): VPA = 23 (47,9%), LVT = 11 (22,9%), VGB = 9 (18,8%), ACTH = 7 (14,6%), PB = 4(8,3%), CBL = 3 (6,3%), TPM = 2 (4,55%), LTG = 1 (2,1%), CLZ = 1(2,1%), OXC = 1 (2,1%). None of the patients was on the ketogenic diet (KD). As a result of treatment, 16 treated patients were seizures free.

EG was divided into 2 subgroups. The epilepsy group with seizures (EG_1_) included 12 patients with seizures in the course of study (median age was 10 months, female to male ratio was 4:8) [Table 1]. Age of epilepsy onset was 1–30 months. Focal onset seizures were classified in 2 patients, generalized onset seizures in 7 patients, focal and generalized onset seizures in 2 patients. Focal epilepsy type was classified in 2 patient, generalized type in 7 patients, combined focal and generalized type in 2 patients, epileptic spasm in 1 patient and epilepsy syndromes in 8 patients (West syndrome in 5, Lennox-Gastaut syndrome in 2, Dravet syndrome in 1). The etiology of epilepsy was established in 6 patients (structural etiology in 5 patients: hypoxic-ischemic encephalopathy in 4 and genetic etiology in 1 patient: Dravet syndrome) and remained unknown in 50% (n = 6). Development delay was identified in 10 patients.

**Table 1:** Clinical characteristics of epilepsy group (EG_1_) with seizures.

No	Gen	Age (month)	OA	Seizures types	Epilepsy types	Epilepsy syndromes	Etiology	AEDs Tried	Co-morbidities
1	M	9	1	Focal	Focal	West syndrome	Structural HIE	LVT, VPA, VGB	DD
2	M	7	5	Generalized	Generalized	Epileptic spasms	Structural HIE	LVT, VPA	DD
3	M	48	6	Focal and Generalized	Combined Focal Generalized	Lennox-Gastaut syndrome	Structural HIE	VPA, LTG, TPM	DD
4	M	7	3	Generelized	Generalized	West syndrome	Unknown	LVT, VPA, ACTH	DD
5	F	30	30	Focal Generalized	Combined Focal Generalized	Dravet syndrome	Genetic	VPA, CLB	
6	M	8	5	Generalized	Generalized	West syndrome	Unknown	VPA, ACTH, VGB	DD
7	F	21	18	Generalized	Generalized		Unknown	VPA	
8	F	7	6	Generalized	Generalized	West syndrome	Unknown	VPA, VGB, ACTH	DD
9	F	30	8	Focal and Generalized	Focal and Generalized	Lennox-Gastaut syndrome	Structural HIE	VPA, PB, CLZ, TPM	DD
10	M	16	14	Generalized	Generalized		Unknown	VPA, LVT	DD
11	M	7	1	Generalized	Generalized		Unknown	LVT, VPA	DD
12	M	11	10	Focal	Focal	West syndrome	Structural HIE	ACTH,VPA	DD

Abbreviation: No – patient number; Gen - gender: male/female (M/F); Age - age at investigation; OA - age of epilepsy onset; HIE - Hipoxic-ischemic encephalopathy; AEDs - Antiepileptic drugs; VPA – Valproic acid; LVT – levetiracetam; VGB – Vigabatrin; ACTH –Adrenocorticotrophic hormone; PB –Phenobarbital; CLB – Clobazam; TPM – Topiramate; LTG -Lamotrigine; CLZ – Clonazepam; DD – developmental delay.

EG_2_ was the subgroup of the patients with no seizures during the study (median age was 13 months, female to male ratio was 9:7, n = 16) [Table 2]. Age of epilepsy onset was 1–36 month. Focal onset seizures were classified in 8 patients, generalized onset seizures in 5 patients, focal and generalized onset seizures in 2 patients. Epilepsy types were classified as: focal epilepsy in 8 patients, generalized epilepsy in 5 patients, combined focal and generalized epilepsy in 2 patients. Epilepsy syndromes was established in 6 patients (West syndrome in 4, Lennox-Gastaut syndrome in 1, Dravet syndrome in 1). The etiology of epilepsy was established in 12 (75%) patients (structural etiology in 10 patients: brain malformation in 6, hamartoma in 1, hypoxic-ischemic encephalopathy in 3, genetic etiology in 6 patient: Noonan syndrome, chromosomal aberration – trisomy 21, TSC, NF1, SCN1A mutation and metabolic etiology in 1 patient: methylomalonic aciduria) and remained unknown in 25% (n = 4). Development delay was identified in 13 patients, ASD in 1 patient.

**Table 2:** Clinical characteristics of epilepsy group (EG_2_) without seizures.

No	Gen	Age (month)	OA	Seizures types	Epilepsy types	Epilepsy syndromes	Etiology	AEDs Tried	Co-morbidities
1	M	12	4	Focal	Focal	West syndrome	Structural (brain malformation) Genetic (Noonan syndrome)	VPA, VGB	DD
2	M	12	7	Generalized	Generalized		Unknown	LVT, VPA	DD
3	F	48	20	Focal	Focal		Structural (brain malformation)	VPA, OXC	ASD, DD
4	F	36	36	Focal	Focal		Unknown	VPA	
5	F	30	15	Generalized	Generalized		Unknown	VPA	DD
6	M	24	10	Focal	Focal		Structural (brain malformation) Genetic (chromosomal abberation Trisomy 21)	VPA, PB	DD
7	M	10	9	Generalized	Generalized		Structural HIE	VPA	
8	F	24	23	Focal	Focal		Structural (hamartoma) Genetic (TSC)	VPA, VGB,	DD
9	F	18	5	Generalized	Generalized		Unknown	VPA,	
10	F	7	4	Focal	Focal	West syndrome	Structural (brain malformation) Genetic (NF1)	VGB, ACTH, LVT	DD
11	F	7	1	Focal	Focal		Structural HIE	ACTH, VGB, LVT, VPA, CLB	DD
12	F	3	1	Focal	Focal		Structural HIE	PB, LVT	DD
13	F	6	5	Generalized	Generalized	West syndrome	Metabolic genetic (MA)	VGB, ACTH	DD
14	M	4	4	Generalized	Generalized	West syndrome	Structural (brain malformation)	VGB, LVT, ACTH	DD
15	M	19	6	Focal and Generalized	Combined Focal Generalized	Dravet syndrome	Genetic	VPA, LVT, CLB	DD
16	M	14	4	Focal and Generalized	Combined Focal Generalized	Lennox-Gastaut s.	Structural HIE	VPA, PB	DD

Abbreviation: No – patient number; Gen – gender: male/female (M/F); Age - age at investigation; OA - age of epilepsy onset; HIE - Hypoxic-ischemic encephalopathy; AEDs - Antiepileptic drugs; VPA – Valproic acid; LVT – levetiracetam; VGB – Vigabatrin; ACTH –Adrenocorticotrophic hormone; PB –Phenobarbital; CLB – Clobazam; OXC – Oxcarbazepine; DD – developmental delay; ASD – autistic spectrum disorders; TSC – tuberous sclerosis complex; NF1 – neurofibromatosis; MA - methylmalonic aciduria.

In all EG patients the concentrations of valproic acid in serum were in the therapeutic range, the patients had normal liver and renal functions and the serum transaminases levels were normal.

The RG group included 20 patients (median age was 20 months, female to male ratio was 7:13). All children revealed no neurological symptoms, no development delay nor chronic diseases. The family and gestation history were negative. No abnormalities were revealed in the routine laboratory tests, EEG and MRI of the brain. All children were drug free.

A relatively broad age span (from 3 to 48 months) of the EG group is caused by the differences in the age of epilepsy onset in the children and the resulting later diagnosis of the refractory epilepsy. However, in order not to reduce the already small group size, the youngest patients were not excluded from the study. On the other hand, since healthy children should not be subjected to research (due to ethical issues), the reference (RG) group was selected from the patients with minor health problems (according to the inclusion criteria described above) diagnosed in our Department. Such diagnostic procedures are particularly difficult under the age of 12 months. Thus, matching both groups on age was a challenge. To diminish the study limitation due to the age mismatch of the studied groups, the age correction was applied to the data, as described in detail in section 2.8.

### Clinical presentations

The clinical characteristics of the EG patients are shown in Tables 1 and 2.

### Serum samples preparation for NMR spectroscopy

Overnight peripheral blood samples were collected between 6 and 9 a.m. In 5 patients, the samples were collected twice: before and after AED modification. The total number of acquired serum samples was 53 (33 in the EG group and 20 in the RG group). The sera samples for the metabolomics experiment were prepared according to the modified Bruker protocol^18^ involving two-step thawing (in 4 °C and at room temperature) and using phosphate buffer (pH 7.4) with D_2_O and TSP. The aliquots of 600 µl of the solution were poured into 5 mm NMR tubes (Wilmad WG-1235-7) and stored at 4 °C until the spectroscopic analysis.

### Measurement protocols

1H NMR spectra were acquired on a Bruker 400 MHz Avance III spectrometer (Bruker Biospin, Rheinstetten, Germany) equipped with a 5 mm PABBI probe. The quality assurance, measurement and the post-processing procedures followed the protocol previously described by Boguszewicz *et al*. in the previous work^18^. Quality assurance tests were carried out in a daily manner prior to the start of the measurements, adjustment of acquisition parameters was always done for each measured sample. Spectra were acquired with constant receiver gain (90.5) and at temperature of 310 K. NOESY (Nuclear Overhauser Effect Spectroscopy), CPMG (Carr-Purcell-Meiboom-Gill), DIFF (diffusion edited) and two dimensional JRES (J-resolved) spectra were acquired for each blood serum sample.

The total number of acquired NMR spectra was 212 (4 spectra per each of 53 samples). Description and justification of the applied NMR acquisition sequences and parameters are available in Supplementary material.

### Spectra post-processing

Exponential line broadening (0.3 Hz) and automatic phase correction were applied using Topspin (Bruker Biospin). The Amix software (Bruker Biospin) was used for alignment of NMR spectra to alanine signal at 1.5 ppm, followed by narrowing of the spectral region to the range between 9.0–0.5 ppm, removal of the residual water signal and spectral bucketing (with bucket size of 0.002 ppm). No normalization was applied. Our previous results show that strict adherence to the sample preparation protocol and the measurement protocol provides very stable and reproducible results allowing for biological interpretations^18–20^.

### Metabolite quantification

1D positive projections of JRES spectra were used for quantification of low molecular weight metabolites, while the lipid signals were quantified based on the diffusion edited spectra. The peaks were integrated by area using AMIX (Bruker Biospin) and the integrals were measured in the spectral regions defined individually for each low molecular weight metabolite, whereas in case of the lipid signals 0.12 ppm range around a particular peak was applied.

### Metabolite identification

Metabolites identification was done based on comparison with reference compounds library (in Chenomx NMR Suite Professional (Chenomx Inc., Edmonton, Canada)), multiplicity and scalar couplings information extracted from 2D JRES spectra as well as information from Human Metabolome Database (http://www.hmdb.ca/) and available literature^21^.

### Age dependency

Because the concentration of many metabolites change during the development, especially in infancy and toddlerhood^22–24^, the integral intensities of the NMR signals were investigated upon their age dependency and corrected using the residual method^25,26^. The residual method is based on determining the course of variation of a feature in the control group and calculating the predicted value of this feature for the entire dataset. The residuals are acquired by subtracting this predicted course from the observed values for each subject and represent the deviations from the course predicted for a control group (using the subject’s specific values for each covariate)^25,26^. The age dependency was determined from the metabolic profiles measured for the reference group – the patients revealing no metabolic disturbances due to epilepsy.

There are many publications that describe the normal spectral appearance and the concentrations of the metabolites in the developing organism. Such reports provide a basis for calculation the differences between the spectral data acquired for children and adults as well as a norm from where to investigate pathology^22–24^. In these works various mathematical functions are used to describe the changes in the metabolic concentrations with age (the monoexponential or multiexponential functions, logarithm and linear regression).

In our approach the percentage of the explained variance and the significance of the parameters of various models in the description of the relationship between metabolite integrals and age (monoexponential, multiexponential, logarithm and homographic function) were analyzed. The homographic function was selected as it describes best the data variability and the desirable traits of monotonicity.

Spearman’s rank correlation coefficient, with standard significance threshold of p < 0.05, was chosen to determine if the nonlinear age-associated changes are observed in the acquired data. The analysis and modeling of the developmental metabolic changes were performed only for the statistically significant age-related correlations.

In accordance with the residual method the age-related dependencies of the metabolite concentrations were approximated by the homographic function C^RG^(age) defined as^27^:

1 $${C}^{RG}(age)={a}_{1}+\frac{{a}_{2}}{age}$$

This is a rational function with a horizontal asymptote for age tending to +∞ equal a_1_, whereas the parameter a_2_ determines the developmental dynamics seen in serum metabolome.

The parameters a_1_ and a_2_, describing the maturation processes, were estimated using the Gauss–Newton algorithm and the nonlinear least squares method.

The statistical validity of the obtained model was checked with the normality test of the residuals and the statistical significance of the estimated parameter, the R-value > 0.45.

The age-related functions reflecting the metabolic changes in RG were assumed as the references course for the residual values calculation to make the level of the metabolomic marker independent of age for the R (R^RG^) and epilepsy (R^EG^) groups^25,26^:

2 $${R}^{RG}={C}_{measured}^{RG}-{C}^{RG}(age),\,{R}^{EG}={C}_{measured}^{EG}-{C}^{RG}(age)$$

where ${C}_{measured}^{RG}$ and ${C}_{measured}^{EG}$ are the metabolite integrals quantified from the NMR spectra.

### Data analysis

Multivariate analyses of the NMR spectra were carried out using SIMCA-P+ (Umetrics, vs. 14) and Stata (StataCorp LP, vs. 13.1) software. The NMR variables were Pareto scaled. The initial analyses were conducted using unsupervised principal component analysis (PCA). Then, orthogonal partial least square discriminant analysis (OPLS-DA) was applied. The results from the multivariate projection techniques (MPT) are presented graphically in two types of plots, which detailed description and interpretation is available in^18^.

Because of the relatively small size of the studied group validation of the supervised OPLS-DA model was carried out using internal cross-validation. The statistical significance of the estimated predictive power of the OPLS-DA model was tested using ANOVA of the cross-validated residuals (cv-ANOVA) test.

The metabolites integrals were evaluated for their statistical significance with t-student test and Mann-Whitney U (MWU) test (for non-normally distributed variables, Shapiro-Wilk).The correlations between age and the metabolites integrals were checked using Spearman’s rank correlation coefficients. Statistica software (Statsoft, v. 12) was applied for univariate statistics.

The final classification model based on the metabolite integrals and age corrected metabolite integrals (R) was built using linear discriminant analysis (LDA) for Z-normalized metabolite data. The LDA method is focused on finding the linear combination of the individual variables that will provide the greatest separation between the groups. The LDA model is based on assumptions that the observations in each group (EG and RG) have a multivariate normal distribution and the covariance matrices are equal across the groups but with different means. The assumptions of multivariate normality of the observation vector and the covariance matrix equality were tested using Doornik-Hansen and F (Box) tests, respectively.

The LDA results are presented graphically and tabulated. The assessment of metabolites importance for discrimination between the EG and RG groups is based on LDA structure coefficients measuring the correlation between the discriminant function and each metabolite. The evaluation of LDA classification performance was carried out using a stepwise approach. First, the predictive power of the LDA model was validated using leave-one-out validation (LOO) using the prior probabilities of 0.53 and 0.47 for the EG and RG groups, respectively. The association between the studied groups is presented by a scores plot, where the LOO discriminant scores (y-scale) are graphically presented for each patients in question (x-scale).

Then, the ROC curve was plotted as a measure for assessing the performance of LDA classifier, which corresponds to the total proportion of the correctly classified observations and the area under curve (AUC).

The metabolic pathway analysis was done using MetaboAnalyst 4.0 (MetPA) with Fisher’s exact test chosen for an over-representation analysis and relative betweenness centrality for pathway topology analysis.

## Results

**Figure 1:**
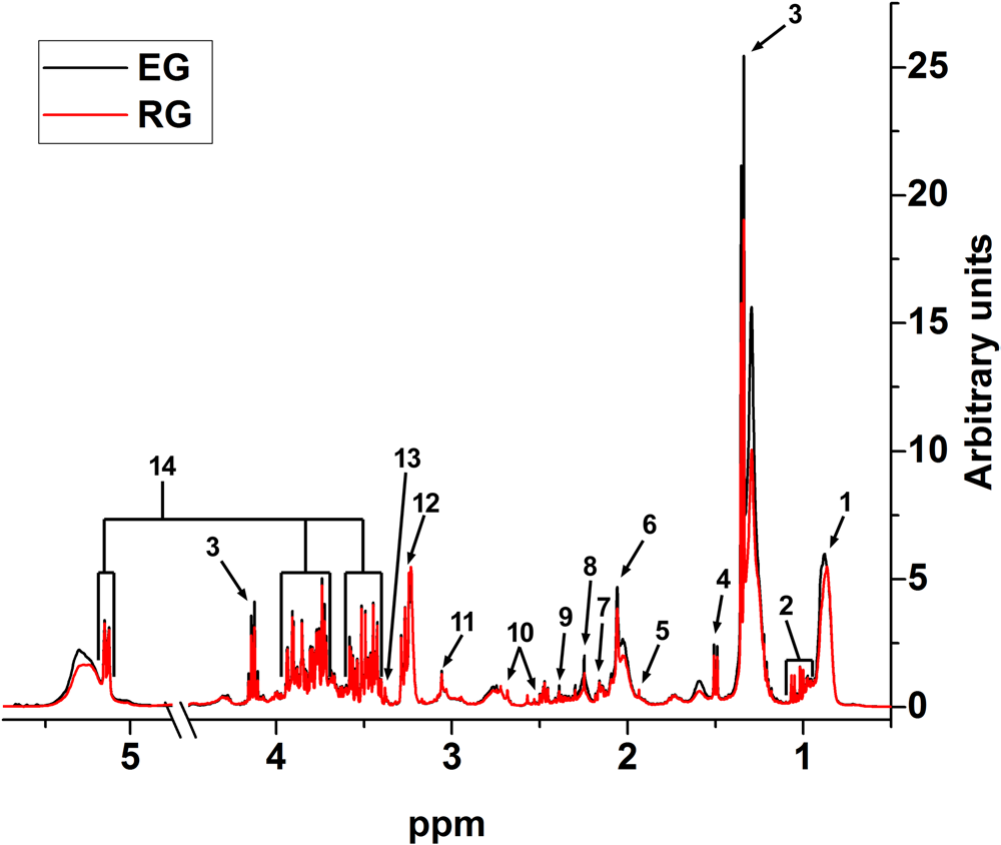
Mean 1H-CPMG spectra of serum samples obtained from the EG and RG groups. Main detected metabolites are indicated: 1, lipids; 2, BCAA (branched chain amino-acids: leucine, isoleucine and valine); 3, lactate; 4, alanine; 5, acetate; 6, N-acetyl-glycoprotein (NAG); 7, glutamine; 8, acetone; 9, pyruvate; 10, citrate; 11, creatinine; 12, choline; 13, methanol; 14, glucose.

Figure 1 shows the mean 1H-CPMG spectra of blood serum obtained from the EG (black) and RG (red) groups – the main metabolites are indicated by numbers.

**Figure 2:**
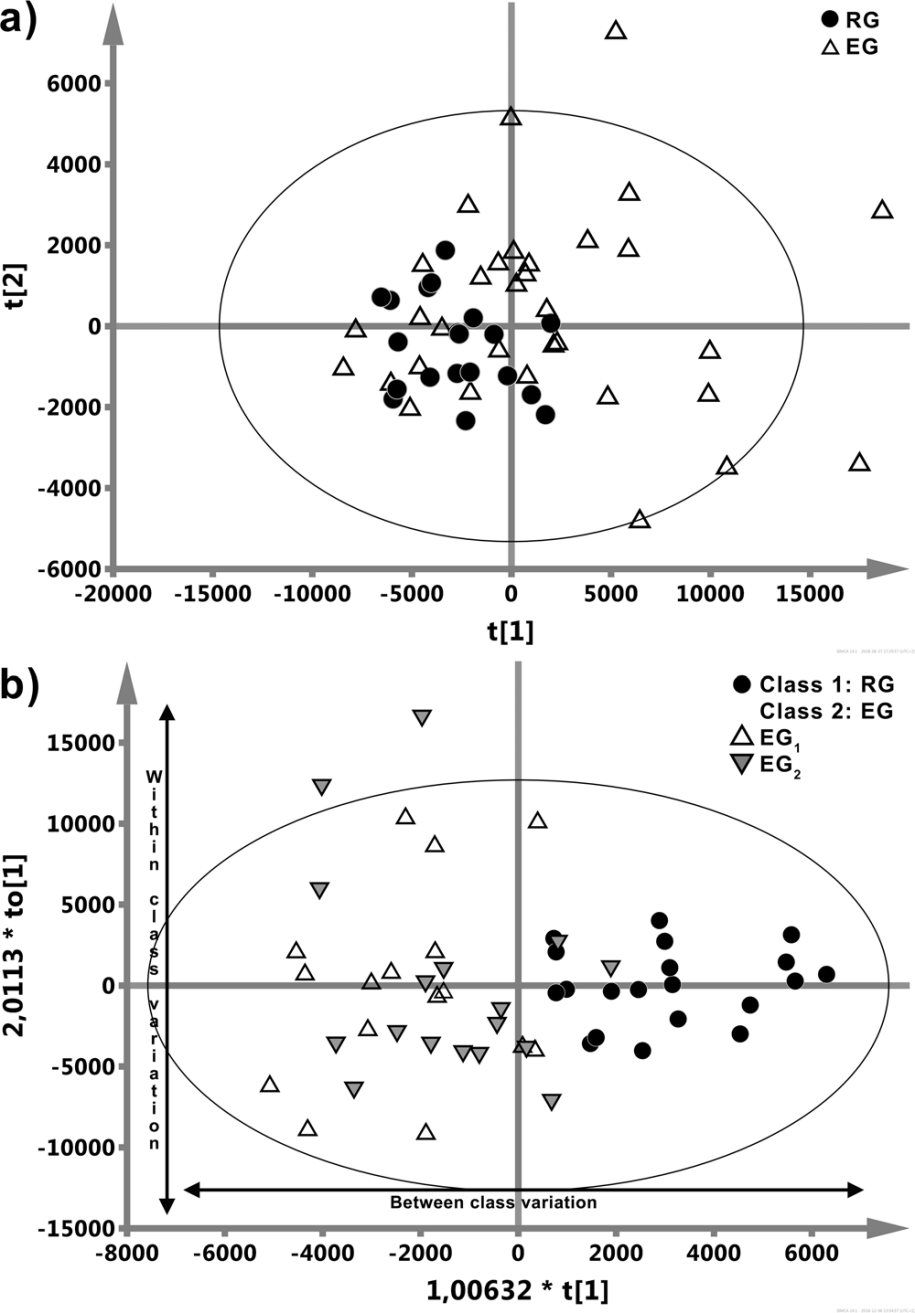
(**a**) PCA analysis of 1 H CPMG NMR serum spectra shows clustering of the epileptic (EG, Δ) and non-epileptic (RG, •) children. (**b**) The R2X scaled (distances in the plot correspond with the explained variation) score plot obtained from the OPLS-DA analysis of 1 H CPMG NMR spectra of serum samples from the patients with epilepsy (EG_1_, Δ, EG_2_,▼) and the reference subjects without epilepsy (RG, •). The information about seizures in EG patients (EG_1_ and EG_2_) was not implemented into the OPLS-DA model and is used purely for a visual assessment of data clustering.

The directions of the largest variation in the four types of the acquired NMR data was visualized using PCA method with the best separation between the EG and RG groups observed in the CPMG spectra. For this reason only the CPMG spectra were subjected to supervised OPLS-DA analyses. The PCA scores plot (Fig. 2a) displays clustering of the epileptic (EG) and non-epileptic (RG) children along the first principal component (t[1]) explaining 56.5% of the variation in the data. The PCA model goodness of fit (R2) and goodness of prediction (Q2) values are 0.815 and 0.693 respectively. In order to identify the epileptic molecular phenotype, two-class OPLS-DA was performed between the RG and EG groups. The OPLS-DA scores plot (Fig. 2b) displays distinct separation between these two classes, along the predictive component (t[1]) representing 15.1% (R2X = 0.151) of the predictive variation - the amount of variation in the data that is correlated to the class separation). The model parameters for the orthogonal variation (R2X(o) – variation in the data uncorrelated (orthogonal) with class separation, R2Y (cum) – total sum of variation related to class separation explained by the model and Q2 – goodness of prediction) were respectively: R2X(o) = 0.423, R2Y (cum) = 0.648 and Q2 = 0.379. The total variation explained by the OPLS-DA model is R2X(cum) = 0.662, p-value of cv-ANOVA < 0.001.

Since the metabolic profiles obtained for EG and RG were found to differ, a possible influence of epileptic seizures occurring prior to blood collection on the metabolic profile of the blood serum was also investigated. There were, however, no statistically significant metabolic differences (OPLS-DA and MWU test) between the EG_1_ and EG_2_ groups. The EG_1_ and EG_2_ groups are denoted in Fig. 2b with various markers, but in view of the statistical results the information about seizures was not implemented into the OPLS-DA model and is used purely for visual assessment of data clustering.

**Figure 3:**
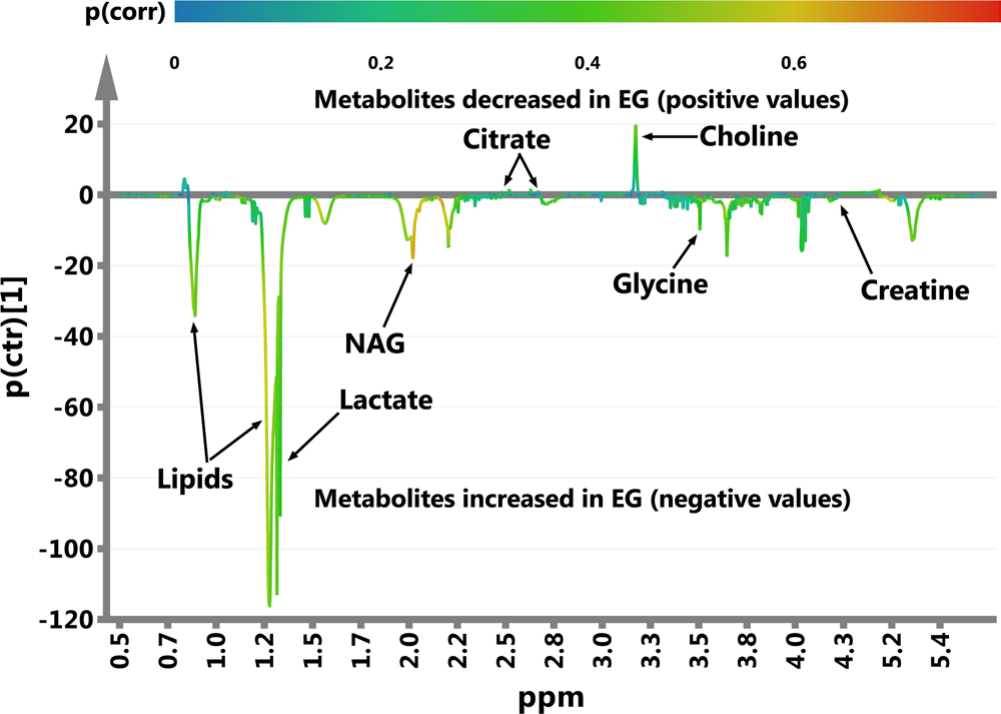
The OPLS-DA s-line plot indicating the metabolites that differentiate the epileptic, EG, and non-epileptic, RG, groups. The correlation of particular metabolites towards segregation between the EG and RG groups (p(corr)) is assessed according to the associated color bar.

The metabolites that discriminate the classes are identified based on the s-line plot from the OPLS-DA analysis (Fig. 3), where the color of the particular spectral points corresponds to their correlation with the class segregation (the more red color, the higher correlation (p(corr)) is)^18^. Metabolomic OPLS-DA analysis of the serum 1H NMR spectra allows identification of the discriminating metabolites (i.e. with p(corr) > 0.3^28^) for the investigated groups. Patients with epilepsy are characterized by the increased serum levels of NAG, lactate, creatine, glycine and lipids (methylene group at 1.3 ppm), whereas the serum levels of citric acid (at 2.55 and 2.7 ppm) and choline are decreased as compared to RG. The OPLS-DA identified discriminating metabolites were quantified and evaluated in terms of their statistical significance with the t-student and MWU tests. Table 3 lists the discriminating metabolites as well as the p values from the statistical significance tests and the p(corr) coefficients values from the OPLS-DA model.

**Table 3:** List of metabolites with age dependent concentrations and discriminant metabolites based on the OPLS-DA analysis.

Metabolites significantly correlated with patients’ age					
No.	Name	Spearman coeff.	Parameter a_1_ of age-related function [arbitrary units]	Parameter a_2_ of age-related function [arbitrary units*month]	
1	Choline	−0.67	26575.93	63438.19	
2	Lactate	−0.46	1.55e + 06	165e + 06	
3	Formate	−0.55	7123.44	26495.39	
4	Dimethylsulfone	0.45	20263.04	−28421.12	
**Metabolites important for discrimination between EG and RG groups**					
	**Name**	**ppm**	**p(corr)**	**p Value**	**p Value (age corrected)**
**Metabolites decreased in EG**					
1	Citrate	2.55/2.7	0.39/0.45	0.0009*/0.0008*	—
2	Choline	3.24	0.49	0.037**	0.7**
**Metabolites increased in EG**					
3	NAG	2.07	0.62	0.018**	—
4	Creatine	3.93	0.41	0.04**	—
5	Lipids	0.9/1.3	0.48/0.56	0.6**/0.023**	—
6	Glycine	3.56	0.67	0.039**	—
7	Lactate	1.33	0.37	0.08**	0.0506**

*t student test, **Mann-Whitney U test.

To identify the potential differences between the EG and RG groups that may be age-dependent, Spearman’s rank correlation coefficients were calculated between age and 27 metabolite integrals (14 metabolites listed in Fig. 1 as well as formate, creatine, glycerol, methanol, phosphocholine, dimethyl sulfone, acetoacetate, 3-hydroxybutyrate, lysine, betaine, threonine, glycine and pyruvate) obtained from the sera average spectrum from the RG group. Three metabolites were found to be significantly negatively correlated (formate, lactate and choline) and one metabolite was positively correlated (dimethylsulfone) with age (Table 3). The significantly age-related metabolites in the RG group were approximated by the homographic function (Eq. 1, Table 3) to obtain the reference metabolome course in the maturation (developmental) process. To remove the age dependency in the concentration of metabolites, the residual values (Eq. 2) were calculated for the EG and RG data. Then, both age-independent and corrected (age-related) data were subjected to statistical analysis. Formate and dimethylsulfone were not important for the class discrimination before and after age-correction. The age-corrected choline became not statistically important while age-corrected lactate became very close to statistical importance with the p value of 0.506 (Table 3).

**Figure 4:**
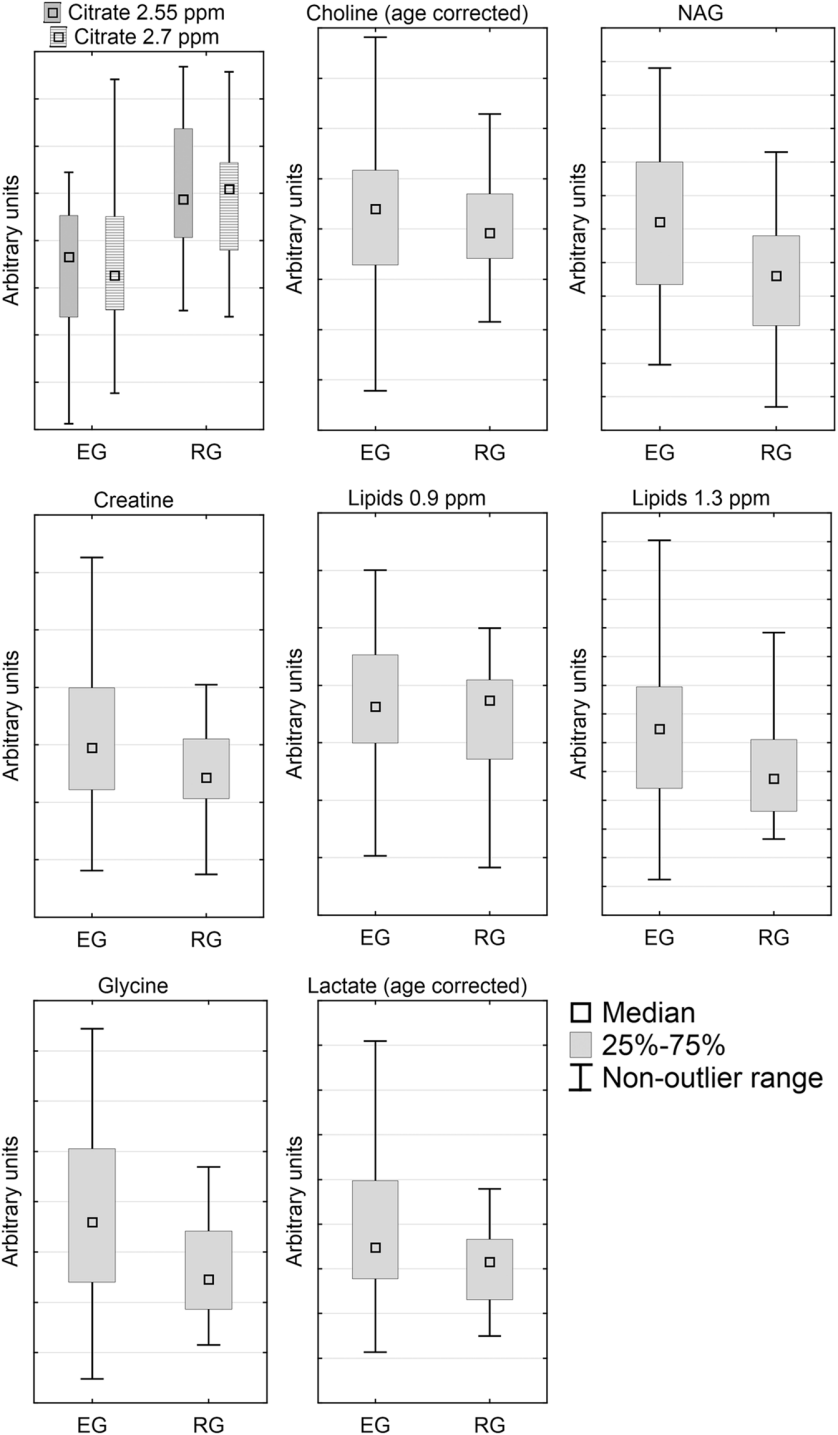
The box plot representation of the relative changes in the significant metabolites identified by the OPLS-DA model as important to discriminate between EG and RG groups. For choline and lactate only the age corrected values are presented.

The box plot representation of the relative changes in the significant metabolites identified by the OPLS-DA model as important to discriminate between EG and RG groups is presented in Fig. 4.

Based on the above results the classification LDA model was created using the significant metabolites (as classification variables) listed in Tables 3 and 4. The model assumption of multivariate normality was fulfilled in RG but not in the EG group. We failed in assessing covariance matrix homogeneity. However, it should be noted that the test statistics for homogeneity of covariance matrix are generally sensitive against to the lack of multivariate normality. Wahl and Kronmal suggest that linear rule for small (or even moderate) samples can potentially result in a greater across-sample stability of the results (with or without normality)^29^. If the nj:p ratios (where nj = number of elements in the group j and p = number of classification variables) are small, then the use of a linear rule is favored even with covariance heterogeneity^30^.

**Table 4:** Results from the LDA analysis.

LDA: structure coefficients for discriminant metabolites			
No.	Metabolites	Structure coefficients	
1	Lactate	0.385	
2	Citrate	−0.557	
3	NAG	0.391	
4	Creatine	0.234	
5	Glycine	0.392	
6	Lipids (signal at 1.3 ppm)	0.390	
LDA leave-one-out (LOO) classification			
True classification	LDA		
	RG	EG	total
Reference group	**16 (80%)**	4 (20%)	20 (100%)
Epilepsy group	7 (25%)	**21 (75%)**	28 (100%)
Total	23 (45,8%)	25 (54,2%)	**48 (100%)**
Priors	0,47	0,53	

**Figure 5:**
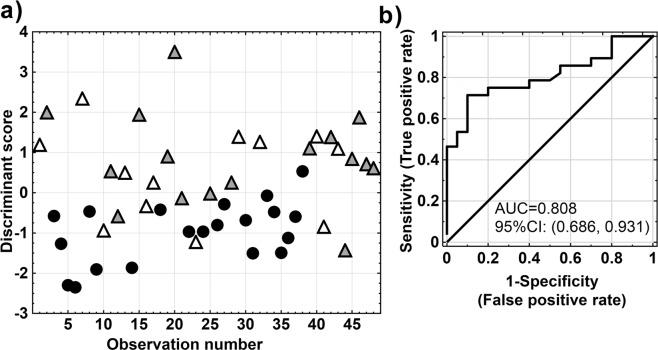
(**a**) Scatter plot of the LOO scores belonging to non-epileptic (RG, •) and epileptic (EG_1_, Δ, EG_2_,▼) children. (**b**) ROC plot for the leave-one-out (LOO) classification model; AUC equals 0.808 and 95%CI is (0.686, 0.931). The proposed threshold was 0.02, and the Youden’s index was 0.61.

The linear discriminant function was calculated for maximal group separation. Table 4 shows the structure coefficients. All metabolites were positively correlated except for citrate (structure coefficient was −0.56). The total leave-one-out (LOO) classification results are included in Table 4. The prior probabilities, on which the classification results partially depend, are also reported in Table 4. The known groups are listed in rows, while the columns correspond to the grouping as assigned by the LDA model. Based on LOO classification true EG (28 observation) had 75% observations correctly classified and 25% observation misclassified. Figure 5 visualizes the LDA model can distinguish between the groups.

The receiver operating characteristic (ROC) plot is useful for evaluation of a biomarker’s ability for classifying the group members and to quantify the diagnostic ability of the LDA model. The maximum potential effectiveness of a biomarker expressed in the Youden Index as well as the area under ROC curve (AUC) being a common numeric summary of the ROC curve are provided in Fig. 5b which shows the ROC curve as a graph of the sensitivity versus 1-specificity of the LOO scores from Fig. 5a. The sensitivity is the fraction of epileptic cases that are correctly classified by the LDA model, whereas the specificity is the fraction of non-epileptic cases that are correctly classified. The prior probability for epileptic cases was 0.53 as presented in Table 4. The global performance of the LDA model in context of ROC analysis is summarized by the AUC. The estimates of AUC (0.808) and 95%CI (0.686, 0.931) match well. The proposed threshold was 0.02.

**Figure 6:**
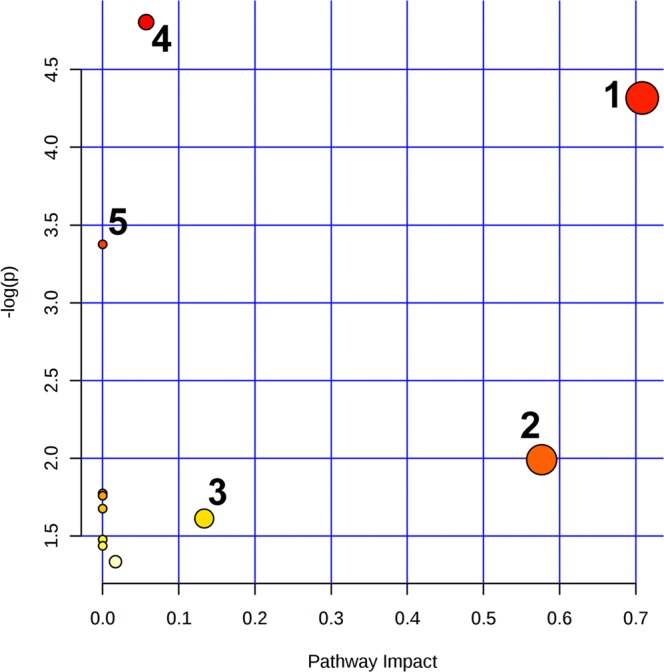
The joint pathway analysis revealing the metabolic pathways altered in the EG group. 1 - glycine serine and threonine metabolism; 2 - citrate cycle (TCA cycle); 3 - glutathione metabolism; 4 - glyoxylate and dicarboxylate metabolism; 5 - cyanoamino acid metabolism.

The joint metabolic pathway analysis showing altered metabolic pathways in the EG group is presented in Fig. 6. The pathways were identified based on significantly altered metabolites listed in Table 3 and 6 (with exception to the lipid metabolites and glycoproteins (NAG)). NAG was implemented into the analysis as gene 1401 corresponding to C-reactive protein (NAG is considered an NMR marker of inflammation). Table 5 summarizes 11 identified metabolic pathways, from which the first 5 were found to be significantly altered in terms of pathway enrichment (p < 0.05) and/or topology (Impact > 0.1): glyoxylate and dicarboxylate metabolism, glycine serine and threonine metabolism, cyanoamino acid metabolism, citrate cycle (TCA cycle) as well as glutathione metabolism. The numbering in Table 5 corresponds to the numbers in Fig. 6.

**Table 5:** The results of the joint pathway analysis from discriminating metabolites between EG and RG groups.

No.	Pathway name	Total	Hits	p-value	−log(p)	Holm p	Impact
1	Glycine serine and threonine metabolism	68	2	**0.013**	4.317	1	**0.708**
2	Citrate cycle (TCA cycle)	50	1	0.136	1.989	1	**0.576**
3	Glutathione metabolism	75	1	0.199	1.611	1	**0.133**
4	Glyoxylate and dicarboxylate metabolism	53	2	**0.008**	4.805	0.654	0.057
5	Cyanoamino acid metabolism	12	1	**0.034**	3.375	1	0
6	Arginine and proline metabolism	102	1	0.263	1.333	1	0.016
7	Primary bile acid biosynthesis	63	1	0.169	1.772	1	0
8	Pyruvate metabolism	64	1	0.172	1.758	1	0
9	Porphyrin and chlorophyll metabolism	70	1	0.187	1.675	1	0
10	Aminoacyl-tRNA biosynthesis	87	1	0.228	1.476	1	0
11	Glycolysis/Gluconeogenesis	91	1	0.237	1.435	1	0

Legend: Total represents the total number of compounds involved in a pathway, Hits is the actual number of matched metabolites in one pathway, Holm p is Holm-Bonferroni adjusted p value and Impact is the pathway impact value from a pathway topology analysis.

## Discussion

The presented study reports significant serum metabolomic differences between the clinically diagnosed young children with drug-resistant epilepsy and the reference non-epileptic individuals between 2–48 months of age. Several statistical and classification methods (supervised and unsupervised) were applied in order to model and explain these metabolic changes. Basic upon them we attempted to create a predictive model for detection of drug resistant-epilepsy in pediatric patients. The factors that might affect the results have been either removed (e.g. age-related differences) or thoroughly discussed (e.g. influence of AEDs) in the section below. Though, there is a relatively small size of the studied group, however due to very young age of the patients it was difficult to gather a larger representative group for a prospective study. For this reason, it was not possible to perform the external validation of the obtained multidimensional models and, instead, the LOO method was used. The predictive linear discriminant analysis model correctly classified 80% (n = 16/20) and 75% (n = 21/28) cases from the RG and EG groups, respectively. Taking into account the extremely complex nature of the data and the enormous weight of the problem, these results seems to be important in terms of the ability of the models build on the NMR serum spectroscopic data to classify the epileptic and non-epileptic young children.

Epileptic seizures are activated by the neuroendocrine system secreting hormones. The hormones induce whole body muscle contractions and increase cardiac, muscular and cerebral oxygen demands, not satisfied by the impaired breathing. Lactate, urea and ammonia are released from strained tissues and, at the same time, creatine kinase and myoglobin leak into bloodstream from exasperated skeletal muscles. This triggers an inflammatory reaction accompanied by cytokine release and leukocytosis^31^. The source of these inflammatory processes is probably the central nervous system, however, they can penetrate from the circulatory system due to breakdown of the blood-brain barrier^32^. The critical inflammatory events, presumably contributing to epilepsy, can be considered a potential source of molecular biomarkers and become targets for therapeutic approaches for epilepsy^33–36^.

The metabolic changes due to generalized tonic clonic seizures, status epilepticus, but even partial seizures are so profound that can be analyzed with laboratory testing of the blood^36,37^. The response of an organism to disease as well as the environmental and treatment-related factors is reflected in the metabolic composition of blood serum, thus the systemic biomarkers, specific for a given epilepsy disease state, can be determined also by metabolomics^38^. Though, the identification of biomarkers of epilepsy has been at the center of interest in the past decade, however the complex and multifactorial nature of epilepsy, and its heterogeneity make this issue still open for further research^35^. Moreover, the number of publications dealing with the quest for biomarkers characteristic for epilepsy in children is sparse.

The present study shows that the serum levels of NAG, creatine, lipids (at 1.3 and 0.9 ppm), glycine and lactate are increased in children with drug-resistant epilepsy, thus these metabolites might be considered as the systemic biomarkers of a diagnostic utility. In case of the lipid signals the statistical significance is reached only for the methylene protons – this so-called “mobile lipids” (ML) signal at 1.3 ppm is due to the fatty acyl chains -CH_2_- in triacylglycerides. Whereas the increase in lactate becomes significant (p = 0.0506) after the correction for age dependency. The level of citric acid (the signals at 2.55 and 2.7 ppm) is significantly decreased in EG as compared to the non-epileptic reference group, whereas the choline changes are not important after the age related correction. In the present study, no significant difference was seen between the EG and RG groups as regards the total glucose levels and there were no significant biomarkers for discrimination of the EG_1_ and EG_2_ groups. The joint pathway analysis revealed 5 significantly altered metabolic pathways, however with only two involved metabolites, glycine (4 pathways) and citrate (2 pathways).

These results should be analyzed taking into account the processes that accompany the epileptic seizures – at the mitochondrial, cellular and systemic levels, as well as the therapeutic process. Mitochondrial dysfunction, seizure induced hypoxia, ROS formation occurring as a result of reoxygenation of the tissue^39^, GABAergic deregulation and inflammation^40^ are among the main processes characteristic for the epilepsy course^41^.

The role of oxidative stress in epilepsies is already well recognized^42^. ROS formation begins with unpaired electrons escaping from the electron transport chain and combining with molecular oxygen, resulting in formation of superoxide. Superoxides easily react with cellular membrane components (i.e. lipids and proteins) and DNA. Hence, the the longer increase in ROS, the higher risk of neurodegeneration, such as that seen in epilepsy^38,43^. This effect depends on age, and presumably, epileptogenesis is strictly associated with mitochondrial dysfunction due to chronic oxidative stress^44^. Liang and Patel^45^ have demonstrated that persistent seizures (status epilepticus) cause oxidative damage to DNA and cellular membrane components leading to protein carbonylation, nitric oxide formation and lipid peroxidation. Though, the levels of oxidative markers were found to be significantly increased in epileptic patients, the antiepileptic drugs were not confirmed to affect the markers’ levels, which suggests that mainly seizures and the induced oxidative stress are responsible for the biomarkers^46^.

In our study the changes in the concentration of lactate, creatine, citrate and lipids may be – to at least some extent – associated with an increased oxidative stress. In tissue hypoxia, impaired mitochondrial oxidation (the major cause for increases in BCAAs levels^47^) results in overproduced and underutilized global and localized lactate. Although we observe increased lactate, there is no evident increase in BCAAs, only elevated plasma glycine is seen in the sera of the EG patients. Glycine, the simplest, nonessential amino acid, is implicated in many biological processes, i.e. the body’s production of DNA, phospholipids and collagen, it is also necessary for glutathione (scavenger of free radicals in nervous system) synthesis, as well as is involved in release of energy. It functions both as an inhibitory and excitatory neurotransmitter and in high concentrations may produce excitoneurotoxicity, seizure and brain damage^48^. Homeostasis of glycine, thus, is important for the maintenance of balance between enhanced and decreased neuronal excitability. The glycine and lactate higher levels in plasma and CSF have been reported in a subgroup of mitochondriopathies related to iron-sulfur cluster defects^49^. Furthermore, glycine serine and threonine metabolism as well as glyoxylate and dicarboxylate metabolism, cyanoamino acid metabolism and glutathione metabolism, where glycine is also involved in, were listed by the joint pathway analysis as significantly altered in EG group. However, a more prosaic reason for the higher glycine levels than for the reference group seems to be also of importance – it may be due to the fact that almost all patients receive the antiepileptic drug valproic acid. VPA is one of the first line drugs for the treatment of all types of epilepsy and its main risk factors include younger age and polytherapy^50^. The inhibitory influence of valproic acid and valproyl‐CoA on the glycine cleavage enzyme complex resulting in elevated amounts of glycine in serum has been known since 1980^51^. Elevated glycine levels in serum were confirmed (along with alanine and serine, however) in epileptic patients treated with VPA alone^52^ and in patients receiving VPA in combination with other AEDs^53^. Significant changes in the serum amino acids laboratory profiles (increased glycine and glutamine and decreased levels of arginine, BCAAs, histidine, methionine, phenylalanine, taurine, threonine and tryptophan) in epileptic patients treated with AEDs were found by Rao *et al*.^54^. However, these changes were not correlated with age, duration of illness or seizure frequency, and seizure type^54^. Their studied group was large (73 epileptic patients), but it was age-heterogeneous, which may matter, especially in case of the comparisons with very young children. Rainesalo *et al*.^55^ also claim that the plasma amino acid levels in epileptic patients may be related to their medication and confirmed that VPA increases the plasma levels of glycine. Although, Scholl-Bürgi *et al*.^56^ using ion exchange chromatography observed no detectable influence of age, gender and AEDs on cerebrospinal fluid/plasma ratios of glycine (as well as of alanine, arginine, histidine, lysine, ornithine, proline and threonine) but in later studies of the homogeneous group of children with propionic acidemia showed that glycine alone is elevated and medication (notably valproic acid) can affect the plasma amino acid concentrations to a variable degree^57^. Thus, our 1H NMR observations generally agree with the mentioned reports, in spite of various analytical techniques used. In our EG group serum glycine is increased and the p value (p = 0.039) is significant in the Mann-Whitney U test.

It is also claimed that VPA therapy impairs lipid metabolism^58^ and may lead to both transient elevation in liver-function tests in 15–30% of patients and a rare, fatal hepatotoxicity^59^. Thus, there are clear and confirmed risk factors for valproic acid-associated hepatotoxicity. One of the markers of the hepatocellular damage are increased blood concentrations of triglycerides^60^. Triglycerides were, in fact, significantly higher in the patients treated with AEDs in the poly therapy regime than in monotherapy one^61^. In our study the methylene CH_2_ lipid signal is markedly higher (the p value equals 0.023) in the patients treated with VPA and additional anticonvulsant drugs than in the reference group.

We observed no statistically significant alternations in other serum amino acid (AA) levels. Such changes are reported in human epilepsy, though the results are often contradictory. In epileptic adults the AA decrease is reported after acute tonic–clonic seizures and partially explained as being a result of increased muscular and metabolic stimulation, similar to that observed in intense training^62,63^. Furthermore, the release of stress hormones during the seizure may also lower the amino acid levels in blood plasma^55^.

Serum of the EG patients had higher levels of creatine/phosphocreatine than in the reference subjects but the differences in the creatinine levels were out of detection. Increased creatine levels were reported in myotonic dystrophy^64^. Creatine and creatinine phosphate are present in blood, muscles and organs, while phosphocreatine is a phosphate donor for the generation of ATP. Creatine is synthesized in liver and kidney and its perturbed level may reflect altered liver functioning. Thus, we can assume that the elevated creatine in blood samples from the EG patients indicate an increased energy demand in the form of ATP and fatty acids, both during the disease and due to impaired liver function caused by VPA. However, this increased level may be also associated – to some extent – with oxidative stress as all creatine kinase isoenzymes are extremely susceptible to oxidative damage^65^.

Another metabolite that plays an important, multidimensional metabolic role is citrate and the fluctuations in the citrate levels are often considered as a useful diagnostic tool or biomarker^66^. Most of the citrate in the blood circulates unbound and the remaining quota is complexed to calcium, potassium and sodium. Citrate molecules are signaling molecules in inflammation processes and in the beginnings of the non-alcoholic fatty liver disease. The citric acid cycle (TCA cycle) or Krebs cycle is the central metabolic process of the cell^67^. Mitochondrial citrate is transported outside the mitochondria by the citrate carrier (CIC), thus, the availability of citrate depends on the amount of citrate transported from the mitochondria, but during the oxidative stress, mitochondrial function is being impaired^68,69^. Citrate, together with glycine, is also involved in glyoxylate and dicarboxylate metabolism which we found significantly altered in terms of the pathway enrichment.

One of the most pronounced differences between the EG and RG groups concerns the NAGs, mainly N-acetylglucosamine and N-acetylneuramic acid – they are acute phase proteins with anti-inflammatory properties and are expressed more during inflammation and immune responses^70,71^. That is why NAG is called an NMR marker of inflammation. Though NAG is higher in children than in adults, the N-acetyl-glycoprotein (NAG) signal at 2.07 ppm in the EG group most presumably reflects the inflammation processes, as it is compared to that for the RG group of the similar age spectrum. Łukasiuk *et al*.^72^ justify the usefulness of proteins reflecting inflammation or neurodegeneration in an epileptic lesion as potential molecular biomarkers. Attractiveness of blood serum and plasma as the sources for such biomarkers is beyond question. However, to date, the only inflammatory proteins proposed as the markers of epileptogenesis are C-reactive protein, interleukin 1-beta, and interleukin 6^72^ – presumably, because their presence or excess in the blood can arise both “spillover” of neuroinflammatory molecules, as well as due to peripheral inflammation.

Similar study was done by Murgia *et al*.^73^ in the adult subjects with epilepsy, however, they excluded the patients receiving valproic acid and lacosamide. Their main findings in the sera of the epileptic, drug resistant adults^73^ are the decreased concentrations of glucose, citrate, and lactate and the increased levels of ketone bodies (3-OH-butyrate, acetate, acetoacetate, and acetone) as compared to the controls. The only common feature is the decreased level of citrate – thus the feature that presumably reflects the oxidative stress. The discrepancy between the metabolic profiles of the adults and children with epilepsy seems, however, to be expected, as the metabolic profiles are age dependent and reflect different aging processes^74^. Furthermore, the age-specificity is also responsible for a different rate of valproate metabolism and thus, a various degree of hepatotoxicity – the elderly may be more vulnerable to adverse effects of AEDs than young children undergoing significant maturation changes.

The main limitation of the study – the relatively small patients groups sizes in the individual age categories – has obviously a deleterious effect on the probability of differentiating the age related effects from those due to epilepsy and makes it difficult to externally validate the multivariate models. Despite these threats, we decided to perform the modeling using the age-corrected data and to strengthen the analysis by applying various multivariate modeling methods. We believe, that our results of the 1H NMR serum studies on epilepsy are important, because there is not much papers on this subject available and there is even less those showing the diagnostic usefulness of the high resolution NMR spectroscopy techniques in epileptic young children. The NMR sera studies and NMR-based metabolomics provide important insights into the underlying epileptic processes and may be useful for understanding the epilepsy and the therapy effects in children.

### Ethical approval

All procedures performed in studies involving human participants were in accordance with the ethical standards of the institutional and/or national research committee and with the 1964 Helsinki declaration and its later amendments or comparable ethical standards.

### Informed consent

Informed consent was obtained from legal representatives of all individual participants included in the study.

## Conclusion

To our best knowledge this is the first attempt of metabolic description of drug-resistant epilepsy in young children and infants performed with the use of 1H NMR-based metabolomics. We identified 6 serum metabolites discriminating the epileptic patients from the reference group. These metabolites are involved in 11 metabolic pathways of which 5 were significantly altered. Although, the interpretation of the results requires verification on a larger group of patients, however, the first guesses indicate the coincidence of oxidative stress (decreased citrate and increased lactate, creatine and lipids), inflammatory state (increased level of N-acetyloglycoprotein), as well as the valproic acid therapy related metabolic disturbances (presumably resulting in elevated glycine) as the characteristic metabolic pattern for pediatric drug-resistant epilepsy. Furthermore, we identified 5 significantly altered metabolic pathways (TCA cycle, glutathione metabolism and several amino acids metabolisms as well as glyoxylate and dicarboxylate metabolism) in the epilepsy group, four of them involving glycine and two involving citrate. The metabolic phenotyping of the therapy course in young epileptic children seems to be useful in providing insight into the mechanisms of treatment-resistance or help in diagnosis.

## Supplementary information

Supplementary material

## Data Availability

The datasets generated during and/or analysed during the current study are available from the corresponding author on reasonable request.
