# Supplementary material for: NMR-based metabolomics in pediatric drug resistant epilepsy – preliminary results
Source: Sci Rep. 2019 Oct 21;9:15035. doi: 10.1038/s41598-019-51337-z (PMC6803684; doi:10.1038/s41598-019-51337-z)
Supplement: Supplementary file 1 — Supplementary material [file 41598_2019_51337_MOESM1_ESM.docx]

**NMR-based metabolomics in pediatric drug resistant epilepsy – preliminary results**

Łukasz Boguszewicz^1,*^, Ewa Jamroz^2^, Mateusz Ciszek^1^, Ewa Emich-Widera^3^, Marek Kijonka^1^, Tomasz Banasik^1^, Agnieszka Skorupa^1^, Maria Sokół^1^

^1^ *Department of Medical Physics, Maria Sklodowska-Curie Memorial Cancer Center and Institute of Oncology, Gliwice Branch, Wybrzeże Armii Krajowej 15, 44-101 Gliwice, Poland*

^2^ *Department of Pediatric and Neurology of Developmental Age: The Independent Public Clinical Hospital No6 of Medical University of Silesia Katowice, Poland*

^3^ *Department of Pediatric Neurology School of Medicine in Katowice , Medical University of Silesia Katowice, Poland*

*Maria Sklodowska-Curie Memorial Cancer Center and Institute of Oncology, Gliwice Branch*

*Street: Wybrzeze Armii Krajowej 15,*

*44-101 Gliwice, Poland.*

* Corresponding author:

Łukasz Boguszewicz

Phone: +48322788047

Fax: +48322313512

[Lukasz.boguszewicz@io.gliwice.pl](mailto:Lukasz.boguszewicz@io.gliwice.pl)

**Acknowledgements**

This work is supported by the Institutional Grand from the Medical University of Silesia, Katowice, Poland, No KNW- 1 -131/N/6/K.

Description and justification of the applied NMR acquisition sequences (experiments):

- NOESY (Nuclear Overhauser Effect Spectroscopy) – to obtain an overview of all types of molecules.
- CPMG (Carr-Purcell-Meiboom-Gill) - to get information on only low molecular weight metabolites.
- DIFF (diffusion edited) - to detect mainly macromolecular signals.
- Two dimensional (2D) JRES (J-resolved) - to visualize scalar couplings and improve metabolite identiﬁcation, while 1D projections of J-resolved spectra were used in data analyses. 1D J-resolved projections show signals from small metabolites, similar to CPMG, but due to homodecoupling each signal appears as a singlet. For any particular 1H resonance, data for all J-couplings appear in the projected 1D JRES spectrum at the same ppm. Removal of J-coupling information from 1D 1H spectra in this way reduces the overlap of resonance peaks from different metabolites, and allows more accurate metabolite quantifications. Therefore such projections are easier to analyze and quantify.

Table S1. NMR pulse sequence parameters.

| Pulse program | **NOESYGPPR1D** | **CPMGPR1D** | **LEDBPGPPR2S1D** | **JRESGPPRQF** |
| --- | --- | --- | --- | --- |
| **TD** | 65536 | 65536 | 65536 | 8192 |
| **SW** [ppm] | 30 | 20 | 30 | 16.62 |
| **AQ** [sec] | 2.73 | 4.09 | 2.73 | 0.62 |
| **D1** [sec] | 4 | 4 | 4 | 2 |
| **D8** [sec] | 0.01 | - | - | - |
| **D16** [sec] | - | - | 0.0002 | 0.0002 |
| **D20** [sec] | - | 0.0003 | 0.12 | - |
| **D21** [sec] | - | - | 0.005 | - |
| **DS** | 4 | 4 | 4 | 16 |
| **L4** | - | 126 | - | - |
| **NS** | 32 | 64 | 64 | 1 |
| **DELTA1** [sec] | - | - | 0.11572488 | - |
| **DELTA2** [sec] | - | - | 0.004172 | - |
